# Sleep Patterns and Cardiovascular Disease Risk: Investigating the Mediating Role of Inflammatory Markers in a Large NHANES Adult Population

**DOI:** 10.1155/mi/3250786

**Published:** 2025-08-21

**Authors:** Xinru Guo, Shiting Mi, Lin Zhao, Tielong Chen

**Affiliations:** Department of Cardiology, Hangzhou TCM Hospital Affiliated to Zhejiang Chinese Medical University, Hangzhou, Zhejiang, China

**Keywords:** cardiovascular disease risk, inflammatory markers, mediating effect, nonlinear relationship, sleep patterns

## Abstract

**Purpose:** Inflammation plays a central role in the occurrence and progression of cardiovascular disease (CVD). However, the intricate relationships between sleep patterns, inflammatory markers, and CVD risk remain insufficiently understood. This study aims to explore these associations, with a specific focus on the mediating role of inflammatory markers and their nonlinear effects.

**Methods:** This study used a multiethnic adult population (*N* = 8752) from the National Health and Nutrition Examination Survey (NHANES) database (four data cycles from 2005–2006, 2007−2008, 2015–2016, and March 2017–2020). Multivariate logistic regression assessed the effects of sleep patterns and inflammatory markers on CVD risk. Furthermore, the mediating effects of the neutrophil–platelet ratio (NPR) and the systemic immune-inflammation index (SII) were assessed. Binary logistic regression was utilized to analyze the correlations between quartile groupings of inflammatory markers (SII and NPR) and CVD, as well as its specific types. The nonlinear relationship between inflammatory markers and CVD risk was explored using a generalized additive model (GAM).

**Results:** Trouble sleep pattern significantly increased the risk of CVD (odds ratio [OR] = 2.7558, 95% confidence interval [CI]: 1.9019, 3.9931, *p*  < 0.0001). Mediation analyses showed NPR mediated 17.38% and SII mediated 3.27% of the association. GAM showed that NPR (*p*-trend < 0.0001) and SII (*p*-trend = 0.001) had a significant inverted “U” shaped relationship with CVD risk, with higher inflammation increasing CVD risk up to a point, after which the risk decreased.

**Conclusion:** Trouble sleep pattern significantly increased CVD risk, NPR and SII partially mediated the relationship between sleep patterns and CVD risk, and the nonlinear relationship with CVD risk suggests a bi-directional mechanism for the regulation of inflammatory markers. These findings offer new insights for inflammatory interventions and sleep health management in CVD prevention.

## 1. Introduction

Cardiovascular disease (CVD) is one of the leading causes of death worldwide, and atherosclerosis is a core pathologic process of CVD that develops through complex mechanisms and is closely related to inflammatory responses [1]. The Multi-Ethnic Study of Atherosclerosis (MESA) is a prospective cohort study designed to noninvasively assess the risk of CVD in ethnically diverse populations [2]. The MESA study recruited more than 6000 men and women from six U.S. communities from a variety of racial and ethnic backgrounds including different racial and ethnic backgrounds, including White, African American, Hispanic, and Chinese. By collecting and analyzing large amounts of data, the MESA study helps shed light on how CVD develops and how risk can be reduced through early detection and intervention [2]. Subclinical vascular composite signs (including coronary artery calcification, carotid plaque, and atherosclerosis) are strong, independent predictors of CVD risk, especially in early asymptomatic individuals [3, 4]. The aim of this study was to utilize the MESA risk score to measure CVD risk.

In contrast to traditional risk factors, such as high blood pressure, high cholesterol, and smoking, the impact of lifestyle factors, particularly sleep patterns, on cardiovascular health has received much attention in recent years. Research has demonstrated that atypical sleep patterns, including sleep deprivation, inadequate sleep quality, or excessively prolonged sleep duration, have been associated with an elevated risk of developing CVD [5–7]. A previous systematic review and meta-analysis by Cappuccio et al. [8] found that sleep deprivation (defined as sleeping less than 6 h per night) was significantly associated with an increased risk of developing CVD, whereas excessive sleep duration (more than 8 h) may also increase the risk [8]. Furthermore, sleep disorders have the potential to impact cardiovascular health through a variety of physiological mechanisms, including dysfunction of the autonomic nervous system [9, 10], activation of the hypothalamic-pituitary-adrenal (HPA) axis [11], and increased oxidative stress [12, 13].

Among these potential mechanisms, inflammatory factors may play a key mediating role in between sleep patterns and CVD risk. Sleep disorders are strongly associated with elevated levels of inflammatory markers [14, 15], which may lead to systemic inflammatory activation [16, 17], and chronic inflammation is an important driver of atherosclerosis and CVD development and progression. In recent years, machine learning and big data analytics have also revealed the key role of oxidative stress and inflammation between sleep disturbances and CVD [12].

Inflammatory markers play an important role in the pathogenesis of CVD. Inflammatory markers, such as C-reactive protein (CRP) and interleukin-6 (IL-6), are closely associated with sleep disorders [18]. The monocyte-to-high-density lipoprotein ratio (MHR) is also recognized as an important marker of inflammation and atherosclerosis, and the MHR has been shown to be a good predictor of the risk of coronary heart disease, heart failure, and other CVD events in patients with obstructive sleep apnea [19–23].

The systemic immune-inflammation index (SII) is a new inflammatory marker based on neutrophil, platelet, and lymphocyte counts, which can effectively and comprehensively reflect the inflammatory state of the body and the status of the immune system. SII has significant correlation with the pathophysiology and prognosis of CVD, and its clinical application in CVD has shown that the SII can be used as an important indicator for the risk assessment and prognosis of CVD [24]. Neutrophil–platelet ratio (NPR) is a widely used marker to assess systemic inflammation, which is usually associated with an increased risk of coronary heart disease [25]. As an inflammatory marker, is closely associated with CVD and sleep disorders, and is a potential predictor of cardiovascular events [26].

However, the role of inflammation in the co-morbidity of sleep disorders and CVD remains incompletely understood. The aim of this study was to investigate the association between sleep patterns, inflammatory markers, and the risk of CVD, and to further examine whether inflammatory markers (SII and NPR) play a mediating role in the relationship between sleep patterns and CVD risk. By systematically analyzing the interrelationships among inflammatory markers, sleep patterns, and CVD risk, this study will provide new perspectives for understanding the effects of sleep on cardiovascular health and provide an innovative theoretical basis for CVD prevention and treatment strategies.

## 2. Materials and Methods

### 2.1. Study Population

Data from the NHANES (National Health and Nutrition Examination Survey) database for the years 2005–2006, 2007−2008, 2015–2016, and March 2017–2020 (*N* = 46,028) were selected for this study. The study population consisted of adults between the ages of 45 and 85 years [27]. NHANES utilizes a complex, multistage probability sampling methodology to obtain a representative sample of individuals comprising the total national population.

A number of inclusion and exclusion criteria were adopted to ensure the accuracy and reliability of the study. Inclusion criteria: adults between the ages of 45 and 85 years. Exclusion criteria: (1) individuals lacking self-reported data on sleep duration or sleep disorders. (2) individuals lacking data on measurements of inflammatory marker levels. (3) individuals lacking a self-reported history of cardiovascular vascular-related diseases. (4) lack of self-reporting by individuals who are taking medications to lower blood pressure and blood lipids. (5) individuals lacking data on key covariates, including level of education, poverty-to-income ratio (PIR), body mass index (BMI), drinking habits, smoking status, waist circumference, hypertension (HTN), and diabetes mellitus (DM). By using these criteria, 8752 individuals were ultimately included, and we aimed to construct a representative and data-complete study sample to more accurately assess the relationship between sleep patterns and CVD risk. The comprehensive consideration of covariates in the study helped to control for potential confounders, thereby more accurately revealing the independent effects of sleep patterns on CVD risk.

### 2.2. Primary Measures

The MESA risk assessment is an important tool. It is used to calculate an individual's risk of major adverse cardiovascular events (MACE) within the next 10 years, which include myocardial infarction (MI), resuscitated cardiac arrest, death from coronary artery disease (CAD), or hemodynamic remodeling with previously or concurrently adjudicated angina pectoris. The algorithm was based on race, sex, age, smoking status (smoking defined as smokers and nonsmokers), diabetes status (diabetes defined as diabetic and nondiabetic patients), the presence of prescription medications for HTN and hyperlipidemia, mean systolic blood pressure (SBP), total cholesterol (TC), high-density lipoprotein cholesterol (HDL-C), and the presence or absence of a history of heart attack in the family (parents, siblings, or children) to assess 10-year CVD risk. We used an overall risk score point based on the above process, with higher scores indicating a higher risk of having CVD, expressed as a MESA risk score percentage (MESA%). Where MESA% is categorized into three risk classes: low (<7.5%), medium (7.5–19%), and high ( ≥20%) [28, 29].

Sleep pattern data were collected using a self-report questionnaire during the health interview. Each participant was asked to answer the computer-assisted personal interview question (SLQ300), “How many hours of sleep do you usually get on a weekday or weekday night? ”. In this study, sleep duration was categorized as short (≤6 h per night), normal (7–9 h per night), or long (>9 h per night). In addition, sleep difficulties were measured by the question (SLQ050), “Have you ever told your doctor that you have sleep problems? “, defining sleep difficulties as yes and no based on the answer. The presence of sleepiness was defined by the answer to the question (SLQ120) “How often do you feel excessively sleepy during the day?” defined by responses to the question, categorized as normal (≤4 times per month) and sleepy (>4 times per month). The generation of an overall sleep pattern score was achieved through the categorization of normal and abnormal factors of sleep behavior as 0 and 1, respectively. The assignment of a score of 1 was determined by the presence of either excessive or inadequate sleep. The summation of the three scores resulted in the identification of healthy (0), moderate (1), and troubled (2–3) sleep patterns [30].

CVD is defined by the following question: “Has a doctor or other health professional ever told you that you have congestive heart failure (CHF)/coronary heart disease (CHD)/angina pectoris/MI/stroke?” Answering yes to any of these questions defines a person as having CVD.

### 2.3. Laboratory Measurements

The Beckman Colter DxH 800 instrument in the NHANES Mobile Examination Center (MEC) was used to measure CBCs on blood specimens and provide blood cell distributions for all participants. For a comprehensive overview of the laboratory methods employed, please refer to the Laboratory Methods Documentation section. The SII is derived by dividing the product of platelet and neutrophil counts by the lymphocyte count ratio. Neutrophil–lymphocyte ratio (NLR) was obtained by calculating the ratio of the neutrophil count to the lymphocyte count. NPR was obtained by the ratio of the neutrophil count to the platelet count. Platelet–lymphocyte ratio (PLR) was obtained by the ratio of the platelet count to the lymphocyte count. The neutrophil–lymphocyte and platelet ratio (NLPR) inflammatory index is obtained by combining the neutrophil, lymphocyte, and platelet counts. Laboratory tests, including glycated hemoglobin (HbA1c), fasting glucose, HDL-C, triglycerides, and low-density lipoproteins (LDL-C), are described in detail on the NHANES website (NHANES homepage).

### 2.4. Covariate

Covariates in this study included demographic characteristics (sex, age, race, educational level, PIR), BMI, weight, height, exercise status, smoking and drinking status, dietary supplement use, self-reported genetic history of familial cardiac disease, DM, and HTN and laboratory findings: white blood cells, lymphocytes, monocytes, neutrophils, platelets, HbA1c, fasting glucose, HDL-C, triglycerides, and LDL-C.

The specific subgroups are as follows: age categories are 45–60 and 61–85. Race categories include Mexican American, Other Hispanic, Non-Hispanic White, Non-Hispanic Black, and Other. Educational level was categorized as less than high school, high school, and more than high school. PIR was calculated by dividing household income by the poverty guideline for a given survey year, and PIR was categorized in this study as 0–1, 1–3, and >3. Weight and height were obtained from a physical examination, and BMI was calculated as the square of body weight in kilograms divided by height in meters. The results were categorized as normal group (<25 kg/m^2^), overweight group (25–30 kg/m^2^), and obese group (≥30 kg / m^2^). Subjects who smoked <100 cigarets in their lifetime were defined as never smokers, those who smoked ≥100 cigarets in their lifetime but did not currently smoke were defined as former smokers, and those who smoked ≥100 cigarets in their lifetime and still smoked were defined as current smokers. Drinking status was categorized as nondrinkers, light to moderate drinkers (0–12 drinks/day), and heavy drinkers (≥12 drinks/day). Exercise status included daily work activities, walking or bicycling activities, and recreational activities, and the number of metabolic equivalent (MET) minutes of physical activity per week was recorded and categorized according to the established criteria: (1) low, < 600 MET min/week; (2) moderate, 600–1200 MET/week; and (3) exceeding, ≥1200 MET min/week [31]. DM was defined as a self-reported history of DM or a documented history of HbA1c ≥6.5%, fasting blood glucose ≥126 mg/dL, oral glucose tolerance test (OGTT) 2 h blood glucose ≥200 mg/dL, and use of hypoglycemic drugs or insulin, with any one of the above being defined as having DM [32]. HTN was defined as a self-reported medical history of HTN, blood pressure levels of ≥140/90 mmHg, or the use of antihypertensive medication, any of which was defined as HTN [33].

### 2.5. Statistical Analysis

All statistical analyses were performed using R4.3.2 software. In descriptive statistics, continuous variables were expressed as means and standard deviations, and categorical variables were expressed as proportions and percentages of the total. For continuous variables, one-way ANOVA was used for comparisons between groups of normally distributed variables, and the Kruskal–Wallis *H* test was used for comparisons between groups of skewed variables. Comparisons between groups of categorical variables were made using the *χ*^2^ test, and *p*-values < 0.05 were considered statistically significant.

The association between sleep patterns and CVD risk was assessed by using multiple logistic regression analysis, and further analyzed using ordered logistic regression (OLR) to calculate the trend *p*-value. Mediation effects were used to analyze whether inflammatory markers mediated the association between sleep patterns and CVD risk. In order to assess the correlation between inflammatory markers and cardiovascular-related diseases, researchers performed a multifactorial binary logistic regression analysis and categorized NPR and SII by quartiles. To better characterize the association between inflammatory markers and MESA risk scores, curve fitting and threshold effect analyses were performed, and the trend *p*-value was calculated.

## 3. Results

### 3.1. Characteristics of Participants

A total of 8752 participants were enrolled in this study, of whom 4474 (51.12%) were female and 4278 (48.88%) were male, with a mean age of 61.1 years (Table 1). Participants were categorized by MESA risk score into three groups: high risk (501, 5.7%), intermediate risk (2660, 30.4%), and low risk (5591, 63.9%). The data showed that participants in the high-risk group were more likely to be male, non-Hispanic white, older, have lower household incomes, less education, and have higher BMI and poorer metabolic health indicators. The high-risk group demonstrated significantly higher levels of cardiovascular-related comorbidities (e.g., coronary heart disease, angina, heart failure, and stroke) as well as inflammation-related metrics (e.g., SII, NLR, and NPR) compared with the low and moderate risk groups. In addition, participants in the high-risk group had poorer sleep patterns and higher rates of abnormal sleep duration, excessive sleepiness, and sleep disorders, suggesting that sleep problems may be one of the important influences on CVD. Other relevant covariates are detailed in Table S1.

### 3.2. Sleep Patterns and Inflammatory Markers Correlate With MESA Risk Levels

As demonstrated in, Table 2 the model that was not adjusted for covariates revealed that trouble sleep patterns significantly elevated the risk of high (odds ratio [OR] = 1.3934, 95% confidence interval [CI]: 1.2356, 1.5715, *p*  < 0.0001) and moderate risk of MESA compared with normal sleep pattern (OR = 1.3309, 95% CI: 1.2460, 1.4216, *p*  < 0.0001). After adjusting for potential confounding variables, the presence of troubled sleeping patterns persisted in its significant association with high-risk outcomes (OR = 2.7558, 95% CI: 1.9019, 3.9931, *p*  < 0.0001) and moderate (OR = 1.6058, 95% CI: 1.3224, 1.9499, *p*  < 0.0001) risks of MESA. In the unadjusted model, intermediate sleep pattern demonstrated a statistically significant association with moderate risk (OR = 1.1883, 95% CI: 1.0860, 1.3003, *p*=0.0002). Nevertheless, following adjustment for potential confounding variables, a persistent statistically significant association with risk was observed in both the high-risk group (OR = 2.4362, 95% CI: 1.4639, 4.0394, *p*=0.0006) and intermediate-risk group (OR = 1.5226, 95% CI: 1.1818, 1.9618, *p*=0.0011).

Analysis of inflammation metrics showed that SII was not significantly associated with risk of MESA in the unadjusted model (*p*=0.5019), but after adjustment for covariates, it was significantly and negatively associated with high-risk MESA (OR = 0.9979, 95% CI: 0.9969, 0.9989, *p*  < 0.0001). NLR significantly reduced the risk of MESA in the unadjusted model (OR = 0.9199, 95% CI: 0.8538, 0.9911, *p*=0.0282), but significantly increased the risk of MESA in the adjusted model (OR = 4.3179, 95% CI: 2.8244, 6.6012, *p*  < 0.0001). PLR was not statistically significant in the unadjusted model but significantly increased high risk of MESA after adjustment (OR = 1.0039, 95% CI: 1.0002, 1.0076, *p*=0.0408).

In addition, NPR significantly increased the risk of high MESA in the unadjusted model (OR = 6.4646, 95% CI: 6.2693, 6.6659, *p*  < 0.0001), and the association was even more pronounced after adjustment for covariates (OR = 13.2173, 95% CI: 9.7151, 17.9821, *p*  < 0.0001). The NLPR has been shown to have a significant impact on the risk in MESA in the unadjusted model (OR = 2.8775, 95% CI: 2.8207, 2.9355, *p*  < 0.0001), however, upon adjusting for the relevant covariates, the NLPR exhibited a tendency toward a substantial reduction in risk (OR = 0.5667, 95% CI: 0.4093, 0.7845, *p*=0.0006). The NPR and NLPR values were log-transformed.

In summary, sleep patterns and some inflammatory indicators (NLR, SII, PLR, NPR, and NLPR) were significantly associated with MESA risk level. Among them, troubled sleep patterns were strongly associated with the risk of MESA, while indicators such as NPR and SII could be used as potential references for assessing the risk of MESA.

In constructing the OLR model, we used the healthy sleep group as the reference group, coded the sleep pattern variable as a dummy variable, and included the other two groups as two dummy variables in the model. Meanwhile, the inflammation index was included as a continuous variable in the regression analysis. The results showed that sleep patterns were linearly associated with cardiovascular risk, meaning that decreased sleep quality may be associated with increased cardiovascular risk. However, the linear trend relationship of the remaining inflammation indicators was not significant, suggesting that the association between inflammation indicators and cardiovascular risk may not be a simple linear pattern.

**Figure 1:**
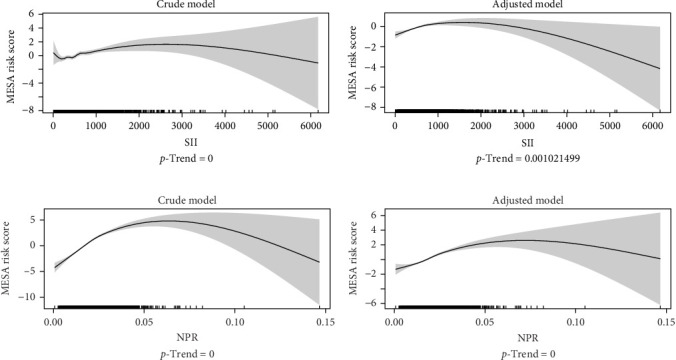
Generalized additive model (GAM) of inflammatory markers and MESA risk score. (A) Relationship between SII and MESA risk score. (B) Relationship between NPR and MESA risk score. Crude models were unadjusted and adjusted models were adjusted for age and sex. NPR, neutrophil–platelet ratio; SII, systemic immune-inflammation index. *p*-trend: trend test based on the GAM model.

To explore whether there is a nonlinear relationship between inflammation and cardiovascular risk, the researchers used a generalized additive model (GAM) in Figure 1 to visualize the association between inflammation and cardiovascular risk. To further validate this relationship, the researchers grouped the inflammatory markers into quartiles and constructed an OLR model to examine the pattern of risk changes at different levels. Group Q1, with the lowest level of inflammation, was used as the reference group, and the remaining three groups, Q2, Q3, and Q4, were included in the model as three dummy variables [34]. Table S2 shows that SII did not have a statistically significant OR in Model 3, but the trend *p*-value was significant (*p*-trend = 0.0309), suggesting a possible overall nonlinear association. In addition, the nonlinear association of NPR with cardiovascular risk further supports that the effect of inflammatory indicators on cardiovascular risk may be more complex.

### 3.3. Mediating Role of Inflammatory Markers on Sleep Patterns and Risk of CVD

**Figure 2:**
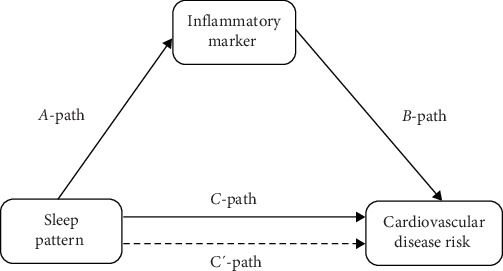
Path diagram of the mediation analysis models.

The mediating effect analysis was performed using the Process 4.0 plug-in developed by Hayes. To investigate whether inflammatory markers mediated the association between sleep patterns and CVD risk, 3 pathways (*a*, *b*, *c*) were used to assess the mediating effect (Figure 2). The following formula was used to calculate the proportion of mediating effects: (mediating effect/total effect) × 100%. The significance level was set at *α* = 0.05, and all tests were two-sided. Significance levels are indicated as *⁣*^*∗∗*^*p* < 0.01, and *⁣*^*∗∗∗*^*p* < 0.001 (Table 3).

As shown in Tables 3 and 4, sleep pattern was positively associated with CVD risk (*p*  < 0.001) and NPR (*p*  < 0.001), and NPR was also positively associated with CVD risk (*p*  < 0.001). The mediating effect was further examined, and the results showed that, after adding NPR, the upper and lower limits of the 95% CI of the direct effect of sleep pattern on CVD risk did not contain 0, and the boot CI was (0.0187, 0.0468). The upper and lower limits of the 95% CI of the mediating effect of NPR did not contain 0, and the boot CI was (0.0036, 0.0105), which means that the mediating effect of NPR is significant and is a partial mediation effect. The direct effect (0.0327) and the mediating effect (0.0069) accounted for 82.62% and 17.38% of the total effect (0.0397), respectively. That is to say, 17.38% of the total association between sleep patterns and 10-year CVD risk was mediated by NPR.

As shown, similarly, sleep pattern was positively associated with CVD risk (*p*  < 0.001) and SII (*p*  < 0.01), and SII was also positively associated with CVD risk (*p*  < 0.001). The mediating effect was further examined, and the results showed that, after the addition of SII, the upper and lower bounds of the 95% CI of the direct effect of sleep pattern on CVD risk did not contain 0, and the boot CI was (0.0240, 0.0527). The upper and lower bounds of the 95% CI of the mediating effect of SII did not contain 0, and the boot CI was (0.0003, 0.0024). The mediation effect of SII is significant and partial. The mediating effect (0.0013) accounted for 3.27% of the total effect (0.0397), thus 3.27% of the total association between sleep patterns and CVD risk was mediated by NPR (Tables 3 and 4).

### 3.4. Correlation of Inflammatory Markers With CVD

The correlation between quartile groupings of inflammatory markers (SII and NPR) and CVDs and their specific types (CHF, coronary heart disease [CHD], angina pectoris, MI, and stroke) was analyzed using binary logistic regression based on the results of Table 5, and the results were as follows:

In the SII subgroup, the risk of CVD was significantly lower in the Q2 group compared with the Q1 group (OR = 0.83, 95% CI: 0.70–0.98, *p*=0.028), whereas there was no significant association with the risk of CVD in the Q3 group (*p*  > 0.05), and the risk of CVD was significantly higher in the Q4 group (OR = 1.28, 95% CI. 1.10–1.50, *p*=0.002). Among specific disease types, the risk of CHF was significantly higher in the Q3 and Q4 groups (OR = 1.45, 95% CI: 1.09–1.93, *p*=0.010 for the Q3 group and OR = 1.63, 95% CI: 1.23–2.16, *p*  < 0.001 for the Q4 group). In addition, the risk of MI was significantly higher in the SII Q4 group (OR = 1.32, 95% CI: 1.04–1.68, *p*=0.025). However, for CHD, angina, and stroke, the association between SII grouping and risk was not significant (*p*  > 0.05).

In the NPR subgroups, the risk of CVD was found to be significantly elevated in the Q2, Q3, and Q4 groups in comparison with the Q1 group. Specifically, the Q2 group exhibited an OR of 1.28 (95% CI: 1.07–1.54, *p*=0.008), the Q3 group demonstrated an OR of 1.71 (95% CI: 1.44–2.05, *p*  < 0.001), and the Q4 group had an OR of 2.71 (95% CI: 2.29–3.20, *p*  < 0.001). For CHF, the Q4 group exhibited a significantly elevated risk with an OR of 4.58 (95% CI: 3.28–6.39, *p*  < 0.001), while the Q3 group also demonstrated an augmented risk (OR = 2.38, 95% CI: 1.66–3.41, *p*  < 0.001). A similar trend was observed for CHD, where the Q4 group exhibited a substantially elevated risk (OR = 3.77, 95% CI: 2.91–4.88, *p*  < 0.001), and the Q3 group demonstrated a notable rise in risk (OR = 1.91, 95% CI: 1.44–2.54, *p*  < 0.001). The risk of angina was also significantly higher in the Q4 group (OR = 3.04, 95% CI: 2.61–4.27, *p*  < 0.001), with the Q3 group showing an increase as well (OR = 1.81, 95% CI: 1.26–2.61, *p*=0.001). For MI, the Q4 group exhibited a significantly elevated risk (OR = 3.12, 95% CI: 2.39–4.06, *p*  < 0.001), with the Q3 group also demonstrating an increased risk (OR = 1.90, 95% CI: 1.44–2.53, *p*  < 0.001). Finally, the Q4 group exhibited a significantly elevated risk of stroke (OR = 1.78, 95% CI: 1.37–2.31, *p*  < 0.001), while the Q3 group demonstrated a moderate increase in risk (OR = 1.45, 95% CI: 1.10–1.90, *p*=0.008).

In summary, the higher quartile groups of both SII and NPR were associated with a significantly higher risk of a variety of CVDs, and the correlation was particularly significant for NPR. Additionally, categorizing the inflammatory markers into tertiles yielded similar conclusions, details of which can be found in Table S3.

### 3.5. Nonlinear Association of Inflammatory Markers With MESA Risk Score

According to the results of the GAM analysis (Figure 1), after adjusting for age and gender, there was a significant nonlinear relationship between SII and MESA risk score (smoothed term degrees of freedom [edf] = 2.599, *p*  < 0.0001), and between NPR and MESA risk score were also significantly nonlinearly related (smoothed term degrees of freedom [edf] = 3.344, *p*  < 0.0001).

The results showed that the effect of SII on MESA risk score tended to increase and then decrease, and this inverted “U” shape relationship was clearly seen in the fitted curves. At low levels of SII, the MESA risk score gradually increases with the increase of SII; however, when SII reaches a certain level, the MESA risk score begins to decrease. In the linear part of the model, age and gender also had a significant effect on MESA risk score. Specifically, for each additional year of age, the MESA risk score increased on average by 0.32 (*p*  < 0.0001). Meanwhile, men had a mean higher MESA risk score of 5.16 (*p*  < 0.0001) compared with women. Overall, the model explained 48.7% of the variance (adjusted *R*^2^ = 0.486) and had a good fit.

The fitted curves showed that the effect of NPR on MESA risk score showed an inverted U-shaped trend: at lower levels of NPR, the MESA risk score gradually increased with the increase of NPR, but the trend began to slow down or even slightly decreased after the NPR reached a certain level. After adjusting for age and gender, age and gender also had a significant effect on the MESA risk score. This was demonstrated by a mean increase of 0.32 (*p*  < 0.0001) in the MESA risk score for each additional year of age. MESA risk scores were, on average 4.90 higher in men compared to women (*p*  < 0.0001). The adjusted *R*^2^ of the model was 0.495, indicating that it explained 49.6% of the variance and provided a better overall fit.

Trend *p*-values and these results suggest that both SII and NPR are nonlinear influences on MESA risk score, while emphasizing the influence of age and gender as important covariates on MESA risk score.

## 4. Discussion

This study examined the association between sleep patterns and CVD risk, and the potential mediating role of inflammatory markers therein, based on a multiethnic sample of adult populations from the NHANES database. Results showed that difficult sleep patterns were significantly associated with increased CVD risk, while inflammatory markers NPR and SII partially mediated the association between sleep patterns and CVD risk. In addition, GAM modeling analysis further revealed that both NPR and SII showed significant nonlinear associations with CVD risk (MESA risk score).

The present study found that troubled sleep pattern significantly increased the risk of CVD, while moderate sleep pattern also showed statistical significance. Mediation analysis showed that NPR mediated 17.38% of the association between sleep patterns and CVD risk (mediation effect = 0.0069, *p*  < 0.001), whereas the mediation effect of SII was weaker, accounting for 3.27% (mediation effect = 0.0013, *p*  < 0.01). There is a complex relationship between sleep patterns and CVD, and poor sleep quality in particular may increase cardiovascular risk through multiple mechanisms [35]. In this study, the presence of a significantly higher level of NPR in patients diagnosed with sleep disorders was identified. This finding may be attributed to immune system dysregulation resulting from sleep deprivation [13]. Numerous studies have previously demonstrated a correlation between NPR and the prognosis of patients diagnosed with acute coronary syndrome (ACS). However, the present study has delved deeper into the association between NPR and atherosclerosis, MI, CHD, and stroke. The present study found that the role of NPR in risk assessment in these diseases was particularly significant, especially in the NPR Q4 group, where the risk of CVD was significantly increased, which is consistent with previous findings [36–38]. SII integrates the relative levels of neutrophils, platelets, and lymphocytes and provides a more complete picture of the impact of inflammation and immune balance on cardiovascular risk. Elevated SII has been found to be strongly associated with the development of ACSs and MI [39], and may influence disease progression through mechanisms such as platelet activation, procoagulant status, and immune imbalance [40]. NPR acts as a predictor of CVD, and the mechanisms of NPR may include endothelial dysfunction through macrophage recruitment, release of arachidonic acid derivatives, superoxide radicals, and adhesion of activated neutrophils to endothelial cells [41]. Insufficient sleep may lead to increased oxidative stress [42], significantly elevated white blood cell counts, and heightened levels of proinflammatory factors [13], causing sustained activation of neutrophils [12], thereby exacerbating the inflammatory response and further contributing to the acceleration of atherosclerosis and impairment of vascular function, thus increasing the risk of CVDs. Inflammation may serve as an important link between sleep and cardiovascular risk [43]. As for the rest of the unexplained portion of about 80% of the total effect, may involve multiple physiological mechanisms and metabolic pathways. Sleep disorders can lead to excessive activation of the sympathetic nervous system, thereby causing increased blood pressure and a faster heart rate [9], which in turn increases the risk of CVD. Additionally, sleep disorders have been associated with metabolic syndrome, as inadequate sleep can result in insulin resistance [44]. Lack of sleep can also lead to increased appetite, energy imbalance, and obesity [45], further increasing the risk of CVD. The association between sleep and CVD may be further exacerbated by psychological factors, such as anxiety and depression, which can act in a synergistic manner.

Analysis of the GAM model showed a significant nonlinear relationship between NPR and SII and MESA risk score, with an inverted “U” curve. At lower levels, elevated NPR and SII significantly increased the MESA risk score, suggesting that inflammatory markers at low to moderate levels may accelerate the progression of atherosclerosis and CVD through inflammation and immune imbalance. At low to moderate levels, the proinflammatory effects of neutrophils may contribute to atherosclerosis formation and plaque instability through the release of reactive oxygen species, enzymes, and cytokines that damage endothelial cells [46]. Elevated platelets further accelerate the progression of arterial lesions by activating procoagulant mechanisms [47]. However, the MESA risk score gradually decreased as NPR and SII exceeded certain levels, suggesting that their effects may be influenced by complex biological regulatory mechanisms or other protective responses. The mechanism for this has not been clarified. High levels of NPR and SII may reflect a higher stage of immune system imbalance (e.g., late stage of severe inflammation or initiation of the body's compensatory mechanisms), thereby reducing the increase in MESA risk score. In the late inflammatory phase, the body may also achieve a balance by down-regulating proinflammatory signals (e.g., NF-*κ*B pathway) and upregulating anti-inflammatory signals [48]. High levels of SII or NPR may reflect the characteristics of this phase rather than the direct proinflammatory effects of peak inflammation. In the hyperinflammatory state, elevated NPR may be associated with immune cell depletion, thus exhibiting features of diminished proinflammatory effects [49]. We can explore the underlying mechanisms of this phenomenon in future research.

In addition, analysis of covariates showed a significant effect of age and gender on MESA risk score. Increasing age accumulates the atherosclerotic burden, further exacerbating the risk of CVD. The higher risk in men compared with women may be associated with hormone levels (e.g., protective effects of estrogen) and a higher burden of traditional cardiovascular risk factors (e.g., smoking and unhealthy diet) [50].

This study systematically explored the mediating role of inflammatory markers between sleep patterns and CVD risk for the first time in a multiethnic adult population. The mediating contributions of NPR and SII were quantified by mediating effects analysis, revealing the bridging role of inflammatory markers to this association. In addition, the study used the GAM to reveal, for the first time, a nonlinear inverted “U” shaped relationship between inflammation markers and CVD risk, providing a mechanism for bidirectional modulation of the effect of inflammation levels on cardiovascular risk across different intervals. This finding expands our understanding of the relationship between sleep disorders, inflammation, and CVD risk, and provides new theoretical basis and clinical perspectives for sleep health management and inflammation intervention strategies.

However, the present study was cross-sectional and therefore causal inferences were limited. It is therefore recommended that future longitudinal studies be conducted in order to reveal the long-term role of NPR and SII in the development of CVD. Secondly, it should be noted that self-reported data on sleep patterns and CVD risk may be subject to bias. The single recordings of inflammatory markers used in this study were limited in their ability to capture dynamic changes, and the potential effects of other factors, such as medication use and acute infections, which were not fully controlled for, should be considered when interpreting the results. Furthermore, the magnitude of the mediation effect indicates that NPR and SII play a partial bridging role between sleep patterns and CVD, but together they account for only about 20.65% of the association; the future remains to be explored further. Although the findings of this study offer a novel perspective on the role of inflammation in the relationship between sleep and CVD, further research is necessary to ascertain their clinical feasibility. Subsequent studies could investigate the clinical application of NPR and SII in patients with sleep disorders to assess their potential in early screening and intervention for reducing CVD risk. This would determine their effectiveness as therapeutic targets and evaluate their potential in early detection and intervention measures.

## 5. Conclusion

The present study demonstrated that difficult sleep patterns were significantly associated with an increased risk of CVD, which was partially mediated by the inflammatory markers NPR and SII. At the same time, NPR and SII showed a significant nonlinear relationship with CVD risk, with an inverted “U” shape. The results of this study provide important evidence for the potential application of sleep health management and inflammation intervention strategies in the prevention of CVD.

## Figures and Tables

**Table 1:** Analysis of basic characteristics and differences.

Variables	Total (*n* = 8752)	Low (*n* = 5591)	Moderate (*n* = 2660)	High (*n* = 501)	*p*-Value
Gender, *n* (%)	—	—	—	—	<0.001
Female	4474 (51.12)	3629 (64.91)	788 (29.62)	57 (11.38)	—
Male	4278 (48.88)	1962 (35.09)	1872 (70.38)	444 (88.62)	—
Age group, *n* (%)	—	—	—	—	<0.001
45–60 years	4105 (46.90)	3526 (63.07)	542 (20.38)	37 (7.39)	—
61–85 years	4647 (53.10)	2065 (36.93)	2118 (79.62)	464 (92.61)	—
Race, *n* (%)	—	—	—	—	<0.001
Mexican American	1062 (12.13)	771 (13.79)	263 (9.89)	28 (5.59)	—
Non-Hispanic Black	1900 (21.71)	1289 (23.05)	537 (20.19)	74 (14.77)	—
Non-Hispanic White	4120 (47.07)	2305 (41.23)	1471 (55.30)	344 (68.66)	—
Other Hispanic	827 (9.45)	593 (10.61)	208 (7.82)	26 (5.19)	—
Other race	843 (9.63)	633 (11.32)	181 (6.80)	29 (5.79)	—
Education, *n* (%)	—	—	—	—	<0.001
Above high school	4824 (55.12)	3289 (58.83)	1315 (49.44)	220 (43.91)	—
Below high school	1824 (20.84)	1042 (18.64)	630 (23.68)	152 (30.34)	—
High school	2104 (24.04)	1260 (22.54)	715 (26.88)	129 (25.75)	—
Sleep time, *n* (%)	—	—	—	—	<0.001
Long	702 (8.02)	383 (6.85)	262 (9.85)	57 (11.38)	—
Normal	5836 (66.68)	3780 (67.61)	1750 (65.79)	306 (61.08)	—
Short	2214 (25.30)	1428 (25.54)	648 (24.36)	138 (27.54)	—
Over sleepy, *n* (%)	—	—	—	—	<0.001
Normal	6951 (79.42)	4533 (81.08)	2044 (76.84)	374 (74.65)	—
Sleepy	1801 (20.58)	1058 (18.92)	616 (23.16)	127 (25.35)	—
Sleep pattern, *n* (%)	—	—	—	—	<0.001
Healthy sleep	3654 (41.75)	2426 (43.39)	1043 (39.21)	185 (36.93)	—
Intermediate sleep	3151 (36.00)	2005 (35.86)	976 (36.69)	170 (33.93)	—
Trouble sleeping	1947 (22.25)	1160 (20.75)	641 (24.10)	146 (29.14)	—
Smoking status, *n* (%)	—	—	—	—	<0.001
Current smoker	1398 (15.97)	761 (13.61)	516 (19.40)	121 (24.15)	—
Former smoker	2719 (31.07)	1485 (26.56)	1009 (37.93)	225 (44.91)	—
Never smoker	4635 (52.96)	3345 (59.83)	1135 (42.67)	155 (30.94)	—
Drinking category, *n* (%)	—	—	—	—	0.105
Heavy drinker	51 (0.58)	26 (0.47)	22 (0.83)	3 (0.60)	—
Low-to-moderate drinker	7463 (85.27)	4744 (84.85)	2285 (85.90)	434 (86.63)	—
Nondrinker	1238 (14.15)	821 (14.68)	353 (13.27)	64 (12.77)	—
Trouble sleeping, *n* (%)	—	—	—	—	0.036
No	6015 (68.73)	3893 (69.63)	1794 (67.44)	328 (65.47)	—
Yes	2737 (31.27)	1698 (30.37)	866 (32.56)	173 (34.53)	—
DM, *n* (%)	—	—	—	—	<0.001
No	6489 (74.14)	4784 (85.57)	1547 (58.16)	158 (31.54)	—
Yes	2263 (25.86)	807 (14.43)	1113 (41.84)	343 (68.46)	—
HTN, *n* (%)	—	—	—	—	<0.001
No	3456 (39.49)	2864 (51.23)	549 (20.64)	43 (8.58)	—
Yes	5296 (60.51)	2727 (48.77)	2111 (79.36)	458 (91.42)	—
SII, mean ± SD	543.13 ± 352.29	525.82 ± 339.27	573.34 ± 369.15	575.87 ± 389.04	<0.001
NPR, mean ± SD	0.02 ± 0.01	0.02 ± 0.01	0.02 ± 0.01	0.02 ± 0.01	<0.001

*Note:* MESA risk levels (Muti-Ethnic Study of Atherosclerosis): low: < 7.5%, moderate: 7.5%–20%, high: > 20%.

Abbreviations: DM, diabetes mellitus; HTN, hypertension; NPR, neutrophil–platelet ratio; SII, systemic immune-inflammation index.

**Table 2:** Associations of sleep pattern with inflammatory markers and MESArisk level (*n* = 8752).

	Crude model							Adjusted model						
	Low		High		Moderate			Low		High		Moderate		
Variables	OR (95% CI)	*p*-Value	OR (95% CI)	*p*-Value	OR (95% CI)	*p*-Value	*p*-Trend	OR (95% CI)	*p*-Value	OR (95% CI)	*p*-Value	OR (95% CI)	*p*-Value	*p*-Trend
Sleep pattern							1.269615e-07							0.003420397
Healthy sleep	ref.		ref.		ref.			ref.		ref.		ref.		
Intermediate sleep	ref.		1.0391 (0.8670, 1.2453)	0.6784	1.1883 (1.0860, 1.3003)	0.0002		ref.		2.4362 (1.4693, 4.0394)	0.0006	1.5226 (1.1818, 1.9618)	0.0011	
Trouble sleep	ref.		1.3934 (1.2356, 1.5715)	<0.0001	1.3309 (1.2460, 1.4216)	<0.0001		ref.		2.7558 (1.9019, 3.9931)	<0.0001	1.6058 (1.3224, 1.9499)	<0.0001	
SII	ref.		0.9998 (0.9993, 1.0004)	0.6061	0.9999 (0.9996, 1.0002)	0.5019	9.118117e-08	ref.		0.9942 (0.9923, 0.9962)	<0.0001	0.9979 (0.9969, 0.9989)	<0.0001	0.2684004
NLR	ref.		0.8801 (0.7745, 1.0002)	0.0504	0.9199 (0.8538, 0.9911)	0.0282	0	ref.		4.3179 (2.8244, 6.6012)	<0.0001	1.9496 (1.4754, 2.5764)	<0.0001	0.7909406
PLR	ref.		1.0003 (0.9978, 1.0029)	0.8053	1.0011 (0.9998, 1.0023)	0.0936	0.009101542	ref.		1.0168 (1.0093, 1.0243)	<0.0001	1.0039 (1.0002, 1.0076)	0.0408	0.789707
NPR	ref.		6.4646 (6.2693, 6.6659)	<0.0001	3.5430 (3.4887, 3.5981)	<0.0001	0	ref.		13.2173 (9.7151, 17.9821)	<0.0001	1.4176 (1.0269, 1.9570)	0.0339	0.9181644
NLPR	ref.		4.6993 (4.5148, 4.8912)	<0.0001	2.8775 (2.8207, 2.9355)	<0.0001	0	ref.		0.0433 (0.0313, 0.0599)	<0.0001	0.5667 (0.4093, 0.7845)	0.0006	0.8619288

*Note:* Both NPR and NLPR were transformed to natural logarithms in the analysis. Crude Model: Unadjusted. Adjusted model: adjust for age, gender, race, education level, physical activity, BMI, waist, height, smoking status, drinking category, HDL, LDL, TCHO, glucose, diabetes, and hypertension. *p*-trend: trend test based on the ordered logistic regression model.

Abbreviations: BMI, body mass index; CI, confidence interval; NLPR, neutrophil–lymphocyte and platelet ratio; NLR, neutrophil–lymphocyte ratio; NPR, neutrophil–platelet ratio; OR, odds ratio; PLR, platelet–lymphocyte ratio; ref, reference; SII, systemic immune-inflammation index.

**Table 3:** Mediation effect of the inflammatory markers on the association between sleep pattern and CVD risk.

Inflammatory marker	Exposure to mediator (*β*)	Mediator to outcome (*β*)	Direct effect	Mediated (indirect) effect	Total effect (exposure to outcome)	Proportion mediated (%)
NPR	0.0001*⁣*^*∗∗∗*^	16.6815*⁣*^*∗∗∗*^	0.0327*⁣*^*∗∗∗*^	0.0069*⁣*^*∗∗∗*^	0.0397*⁣*^*∗∗∗*^	17.38
SII	4.3230*⁣*^*∗∗*^	0.0001*⁣*^*∗∗∗*^	0.0384*⁣*^*∗∗∗*^	0.0013*⁣*^*∗∗∗*^	0.0397*⁣*^*∗∗∗*^	3.27

*Note:* Exposure: sleep patterns; outcome: CVD (cardiovascular disease) risk; mediator: NPR; SII; *⁣*^*∗∗*^*p*  < 0.01; *⁣*^*∗∗∗*^*p*  < 0.001.

Abbreviations: NPR, neutrophil–platelet ratio; SII, systemic immune-inflammation index.

**Table 4:** Inflammatory markers mediating the association between sleep pattern and CVD risk.

Inflammatory marker	Effect	Boot standard error	Boot CI; lower	Boot CI; upper	Proportion mediated (%)
NPR					
Total effect	0.0397	0.0073	0.0253	0.0540	—
Direct effect	0.0327	0.0072	0.0187	0.0468	—
Indirect effect	0.0069	0.0017	0.0036	0.0105	17.38
SII					
Total effect	0.0397	0.0073	0.0253	0.0540	—
Direct effect	0.0384	0.0073	0.0240	0.0527	—
Indirect effect	0.0013	0.0005	0.0003	0.0024	3.27

Abbreviations: NPR, neutrophil–platelet ratio; SII, systemic immune-inflammation index.

**Table 5:** Correlation of inflammatory markers with CVD.

Variables	OR (95% CI)	*p*-Value	OR (95% CI)	*p*-Value	OR (95% CI)	*p*-Value	OR (95% CI)	*p*-Value
	**SII** **Q1**		**SII** **Q2**		**SII** **Q3**		**SII** **Q4**	

CVD	ref.		0.83 (0.70, 0.98)	0.028	1.10 (0.94, 1.30)	0.222	1.28 (1.10, 1.50)	0.002
CHF	ref.		0.84 (0.61, 1.16)	0.286	1.45 (1.09, 1.93)	0.010	1.63 (1.23, 2.16)	<0.001
CHD	ref.		0.91 (0.71, 1.16)	0.440	1.22 (0.97, 1.54)	0.087	1.23 (0.98, 1.56)	0.079
Angina	ref.		0.76 (0.55, 1.05)	0.76	1.07 (0.79, 1.45)	0.652	1.09(0.81, 1.48)	0.564
MI	ref.		0.93 (0.72, 1.20)	0.559	1.16 (0.90, 1.48)	0.246	1.32 (1.04, 1.68)	0.025
Stroke	ref.		0.78 (0.60, 1.02)	0.066	1.04 (0.81, 1.34)	0.745	1.23 (0.97, 1.57)	0.090

	**NPR** **Q1**		**NPR** **Q2**		**NPR** **Q3**		**NPR** **Q4**	

CVD	ref.		1.28 (1.07, 1.54)	0.008	1.71 (1.44, 2.05)	<0.001	2.71 (2.29, 3.20)	<0.001
CHF	ref.		1.47 (0.99, 2.16)	0.053	2.38 (1.66, 3.41)	<0.001	4.58 (3.28, 6.39)	<0.001
CHD	ref.		1.24 (0.91, 1.68)	0.169	1.91 (1.44, 2.54)	<0.001	3.77 (2.91, 4.88)	<0.001
Angina	ref.		1.49 (1.02, 2.18)	0.038	1.81 (1.26, 2.61)	0.001	3.04 (2.61, 4.27)	<0.001
MI	ref.		1.19 (0.87, 1.61)	0.277	1.90 (1.44, 2.53)	<0.001	3.1192.39, 4.06)	<0.001
Stroke	ref.		1.29 (0.998, 1.71)	0.069	1.45(1.10, 1.90)	0.008	1.78 (1.37, 2.31)	<0.001

*Note*: Adjusted for age category, gender.

Abbreviations: CHD, coronary heart disease; CHF, congestive heart failure; CVD, cardiovascular disease; CVD, cardiovascular disease; MI, myocardial infarction.

## Data Availability

This study used the data from a publicly available dataset. These data can be found at https://www.cdc.gov/nchs/nhanes.

## Supporting Information

Supporting Information Table S1. Supplementation of the analysis of basic characteristics and differences. Table S2. Ordered logistic regression (OLR). Table S3. Correlation of inflammatory markers with cardiovascular disease and its classification (tertile classification).
