# Supplementary material for: Sleep Patterns and Cardiovascular Disease Risk: Investigating the Mediating Role of Inflammatory Markers in a Large NHANES Adult Population
Source: Mediators Inflamm. 2025 Aug 21;2025:3250786. doi: 10.1155/mi/3250786 (PMC12393959; doi:10.1155/mi/3250786)
Supplement: Supporting Information — Table S1. Supplementation of the analysis of basic characteristics and differences. Table S2. Ordered logistic regression (OLR). Table S3. Correlation of inflammatory markers with cardiovascular disease and its classification (tertile classification). [file 3250786.f1.docx]

***Supplementary Material***

Sleep Patterns and Cardiovascular Disease Risk: Investigating the Mediating Role of Inflammatory Markers in a Large NHANES Adult Population

**Xinru Guo^a^, Shiting Mi^a^, Lin Zhao^b^, Tielong Chen^b*^**

**Supplementary Table S 1.** Supplementation of the analysis of basic characteristics and differences.

**Supplementary Table S *2.*** Ordered logistic regression (OLR).

**Supplementary Table S 3.** Correlation of inflammatory markers with cardiovascular disease and its classification (tertile classification).**Supplementary Table 1.** Supplementation of the analysis of basic characteristics and differences.

| Variables | Total  (n = 8752) | Low  (n = 5591) | Moderate  (n = 2660) | High  (n = 501) | *P* | |
| --- | --- | --- | --- | --- | --- | --- |
| PIR, n (%) |  |  |  |  | **<.001** | |
| >3 | 3826 (43.72) | 2681 (47.95) | 1003 (37.71) | 142 (28.34) |  | |
| 0-1 | 1307 (14.93) | 820 (14.67) | 401 (15.08) | 86 (17.17) |  | |
| 1-3 | 3619 (41.35) | 2090 (37.38) | 1256 (47.22) | 273 (54.49) |  | |
| BMI, n (%) |  |  |  |  | **<.001** | |
| Normal | 2062 (23.56) | 1445 (25.85) | 533 (20.04) | 84 (16.77) |  | |
| Obesity | 3604 (41.18) | 2239 (40.05) | 1149 (43.20) | 216 (43.11) |  | |
| Overweight | 3086 (35.26) | 1907 (34.11) | 978 (36.77) | 201 (40.12) |  | |
| Met, n (%) |  |  |  |  | 0.815 | |
| Exceed | 3689 (60.85) | 2450 (60.88) | 1055 (60.32) | 184 (63.67) |  | |
| Low | 1319 (21.76) | 882 (21.92) | 380 (21.73) | 57 (19.72) |  | |
| Moderate | 1054 (17.39) | 692 (17.20) | 314 (17.95) | 48 (16.61) |  | |
| CVD, n (%) |  |  |  |  | **<.001** | |
| CVD | 1412 (16.13) | 463 (8.28) | 731 (27.48) | 218 (43.51) |  | |
| non-CVD | 7340 (83.87) | 5128 (91.72) | 1929 (72.52) | 283 (56.49) |  | |
| Dietary Supplements Use, n(%) |  |  |  |  | 0.549 | |
| Yes | 5583 (63.79) | 3555 (63.58) | 1716 (64.51) | 312 (62.28) |  | |
| No | 3169 (36.21) | 2036 (36.42) | 944 (35.49) | 189 (37.72) |  | |
| Family History Heart Disease, n(%) |  |  |  |  | **<.001** | |
| No | 7461 (85.25) | 4991 (89.27) | 2149 (80.79) | 321 (64.07) |  | |
| Yes | 1291 (14.75) | 600 (10.73) | 511 (19.21) | 180 (35.93) |  | |
| Congestive heart failure, n (%) |  |  |  |  | **<.001** | |
| No | 8354 (95.45) | 5472 (97.87) | 2455 (92.29) | 427 (85.23) |  | |
| Yes | 398 (4.55) | 119 (2.13) | 205 (7.71) | 74 (14.77) |  | |
| Coronary heart diseases, n (%) |  |  |  |  | **<.001** | |
| No | 8159 (93.22) | 5452 (97.51) | 2332 (87.67) | 375 (74.85) |  | |
| Yes | 593 (6.78) | 139 (2.49) | 328 (12.33) | 126 (25.15) |  | |
| Angina pectoris, n (%) |  |  |  |  | **<.001** | |
| No | 8422 (96.23) | 5485 (98.10) | 2490 (93.61) | 447 (89.22) |  | |
| Yes | 330 (3.77) | 106 (1.90) | 170 (6.39) | 54 (10.78) |  | |
| Myocardial infarction, n (%) |  |  |  |  | **<.001** | |
| No | 8212 (93.83) | 5452 (97.51) | 2367 (88.98) | 393 (78.44) |  | |
| Yes | 540 (6.17) | 139 (2.49) | 293 (11.02) | 108 (21.56) |  | |
| Stroke, n (%) |  |  |  |  | **<.001** | |
| No | 8248 (94.24) | 5406 (96.69) | 2406 (90.45) | 436 (87.03) |  | |
| Yes | 504 (5.76) | 185 (3.31) | 254 (9.55) | 65 (12.97) |  | |
| Weight, Mean ± SD | 102.38 ± 15.36 | 100.21 ± 15.45 | 105.81 ± 14.45 | 108.42 ± 13.97 | **<.001** | |
| Height, Mean ± SD | 166.47 ± 10.00 | 165.03 ± 9.91 | 168.73 ± 9.80 | 170.51 ± 8.63 | **<.001** | |
| Glycohemoglobin%, Mean ± SD | 5.99 ± 1.14 | 5.80 ± 0.99 | 6.28 ± 1.29 | 6.56 ± 1.26 | **<.001** | |
| Triglyceride (mg/dl), Mean ± SD | 126.90 ± 98.49 | 114.83 ± 84.47 | 142.88 ± 109.44 | 180.24 ± 145.75 | **<.001** | |
| Glucose (mg/dl), Mean ± SD | 117.12 ± 40.17 | 109.62 ± 31.22 | 128.88 ± 49.74 | 139.96 ± 49.38 | **<.001** | |
| TCHO (mg/dl), Mean ± SD | 195.87 ± 42.69 | 198.11 ± 38.69 | 191.02 ± 46.56 | 196.70 ± 58.74 | **<.001** | |
| HDL (mg/dl), Mean ± SD | 54.77 ± 16.78 | 58.05 ± 17.42 | 50.00 ± 13.97 | 43.49 ± 11.45 | **<.001** | |
| LDL (mg/dl), Mean ± SD | 82.37 ± 41.22 | 85.09 ± 39.47 | 78.44 ± 43.06 | 72.89 ± 46.96 | **<.001** | |
| WBC (1000cells/uL) Mean ± SD | 7.14 ± 4.98 | 6.97 ± 5.67 | 7.39 ± 2.85 | 7.86 ± 5.46 | **<.001** | |
| PLT (1000 cells/uL), Mean ± SD | 246.12 ± 66.68 | 253.14 ± 67.17 | 235.51 ± 63.82 | 224.05 ± 63.87 | **<.001** | |
| NEU (1000 cells/uL), Mean ± SD | 4.14 ± 1.63 | 3.99 ± 1.61 | 4.39 ± 1.64 | 4.58 ± 1.62 | **<.001** | |
| LYM (1000 cells/uL), Mean ± SD | 2.17 ± 4.23 | 2.20 ± 4.87 | 2.10 ± 1.99 | 2.31 ± 5.10 | 0.464 | |
| NLR, Mean ± SD | 2.20 ± 1.22 | 2.06 ± 1.14 | 2.42 ± 1.31 | 2.56 ± 1.35 | | **<.001** |
| PLR, Mean ± SD | 130.63 ± 56.65 | 131.69 ± 55.33 | 129.62 ± 59.44 | 124.12 ± 55.59 | | **0.009** |
| NLPR, Mean ± SD | 0.01 ± 0.01 | 0.01 ± 0.01 | 0.01 ± 0.01 | 0.01 ± 0.01 | | **<.001** |
| MHR, Mean ± SD | 0.01 ± 0.01 | 0.01 ± 0.01 | 0.02 ± 0.01 | 0.02 ± 0.01 | | **<.001** |
| CRP (mg/dl), M (Q1, Q3) | 0.04(0.01,0.15) | 0.04(0.01,0.14) | 0.05(0.02,0.17) | 0.05(0.02,0.21) | | **<.001** |

Abbreviations: PIR, poverty/income ratio; BMI, body mass index; CVD, cardiovascular disease, WBC, white blood cells; PLT, platelets; NEU, neutrophils; LYM, Lymphocyte; NLR, neutrophil–lymphocyte ratio; PLR, platelet–lymphocyte ratio; NLPR, Neutrophil-Lymphocyte and Platelet Ratio; MHR, Monocyte-to-HDL-C Ratio; CRP, C-reactive protein.; MESA risk levels（Muti-Ethnic Study of Atherosclerosis）: low:<7.5%; moderate:7.5%-20%, high:>20%.

**Supplementary Table 2.**Ordered logistic regression (OLR)

| Variable | Q1 | Q2 | Q3 | Q4 | P-trend^2^ |
| --- | --- | --- | --- | --- | --- |
| SII | | | | | |
| Median | 256 | 398 | 547 | 861 |  |
| Model 1 | ref. | 1.06 (0.93 ~1.19) | 1.17 (1.04 ~1.33) | 1.43 (1.26 ~1.61) | 7.377681e-10 |
| Model 2 | ref. | 1.12 (0.97 ~1.30) | 1.18 (1.02 ~1.36) | 1.47 (1.28 ~1.69) | 4.313088e-09 |
| Model 3 | ref. | 0.96 (0.76 ~1.20) | 0.88 (0.70 ~1.11) | 1.15 (0.92 ~1.45) | 0.03097814 |
| NPR^3^ | | | | | |
| Median | -3.9 | -3.71 | -3.55 | -3.33 |  |
| Model 1 | ref. | 1.41 (1.24 ~1.62) | 2.25 (1.98 ~ 2.57) | 3.20 (2.82 ~ 3.64) | 0 |
| Model 2 | ref. | 1.18 (1.01 ~1.37) | 1.75 (1.51 ~2.03) | 2.02 (1.74 ~2.33) | 0 |
| Model 3 | ref. | 1.22 (0.96 ~ 1.53) | 1.52 (1.20 ~ 1.92) | 1.41 (1.11 ~ 1.79) | 2.10725e-09 |

^1^ORs and 95%CIs were calculated with the use of the OLR model. Model 1: Unadjusted. Model 2: Adjust for age, gender. Model 3: Adjust for age, gender, race, education level, BMI, family history heart disease, dietary supplements use, smoking status, drinking category, HDL, LDL, TCHO, glucose, Glycohemoglobin, Triglyceride, diabetes and hypertension.

^2^Test for trend based on variables containing the median value of each quartile.

^3^NPR was transformed to natural logarithms in the analysis.

**Supplementary Table 3.** Correlation of inflammatory markers with cardiovascular disease and its classification (tertile classification).

|  | OR (95% CI) | P-value | OR (95% CI) | P-value | OR (95% CI) | P-value |  |
| --- | --- | --- | --- | --- | --- | --- | --- |
|  | SII  Q1 | | SII  Q2 | | SII  Q3 | |  |
| CVD | 1.08 (0.93,1.25) | 0.321 | ref. |  | 1.38 (1.20,1.59) | **<0.001** |  |
| CHF | 0.84 (0.64,1.10) | 0.207 | ref. |  | 1.38 (1.09, 1.76) | **0.008** |  |
| CHD | 0.97 (0.78,1.21) | 0.801 | ref. |  | 1.16 (0.94,1.43) | 0.156 |  |
| Angina | 1.15 (0.87,1.53) | 0.322 | ref. |  | 1.37(1.04,1.80) | **0.023** |  |
| MI | 0.85 (0.68,1.07) | 0.163 | ref. |  | 1.10 (0.89,1.36) | 0.355 |  |
| Stroke | 1.14 (0.91,1.44) | 0.258 | ref. |  | 1.41 (1.13,1.76) | **0.002** |  |
|  | NPR  Q1 |  | NPR  Q2 |  | NPR  Q3 |  |  |
| CVD | 0.79 (0.67,0.92) | **0.003** | ref. |  | 1.60 (1.40, 1.84) | **<0.001** |  |
| CHF | 0.55 (0.40,0.77) | **<0.001** | ref. |  | 1.89 (1.50, 2.39) | **<0.001** |  |
| CHD | 0.74 (0.57,0.95) | **0.017** | ref. |  | 1.76 (1.44, 2.15) | **<0.001** |  |
| Angina | 0.64 (0.47, 0.88) | **0.007** | ref. |  | 1.47 (1.14,1.89) | **0.003** |  |
| MI | 0.87 (0.67,1.12) | 0.288 | ref. |  | 1.76 (1.43,2.18) | **<0.001** |  |
| Stroke | 0.81 (0.64,1.03) | 0.086 | ref. |  | 1.23 (0.99,1.52) | 0.057 |  |

Abbreviations: CVD, cardiovascular disease, CHD, Coronary heart disease, CVD, cardiovascular disease, CHF, Congestive heart failure, MI, Myocardial infarction;

*Adjusted for age category, gender.
